# Museum samples reveal rapid evolution by wild honey bees exposed to a novel parasite

**DOI:** 10.1038/ncomms8991

**Published:** 2015-08-06

**Authors:** Alexander S. Mikheyev, Mandy M. Y. Tin, Jatin Arora, Thomas D. Seeley

**Affiliations:** 1 Okinawa Institute of Science and Technology, 1919-1 Tancha, Onna-son, Kunigami-gun 904-0412, Japan; 2 Research School of Biology, Australian National University, Canberra, Australian Capital Territory 0200, Australia; 3 Department of Paediatrics and Adolescent Medicine, Medical University of Vienna, Lazarettgasse 14, AKH BT 25.3, 1090 Vienna, Austria; 4 Department of Neurobiology and Behavior, Cornell University, Ithaca, New York 14853, USA

## Abstract

Understanding genetic changes caused by novel pathogens and parasites can reveal mechanisms of adaptation and genetic robustness. Using whole-genome sequencing of museum and modern specimens, we describe the genomic changes in a wild population of honey bees in North America following the introduction of the ectoparasitic mite, *Varroa destructor*. Even though colony density in the study population is the same today as in the past, a major loss of haplotypic diversity occurred, indicative of a drastic mitochondrial bottleneck, caused by massive colony mortality. In contrast, nuclear genetic diversity did not change, though hundreds of genes show signs of selection. The genetic diversity within each bee colony, particularly as a consequence of polyandry by queens, may enable preservation of genetic diversity even during population bottlenecks. These findings suggest that genetically diverse honey bee populations can recover from introduced diseases by evolving rapid tolerance, while maintaining much of the standing genetic variation.

## Abstract (web summary)

Introduction of pathogens can cause colony collapse in honey bees. Here, the authors use museum specimens to show widespread colony mortality but unaffected nuclear genetic diversity in a wild population of honey bees in North America following the introduction of ectoparasitic *Varroa* mites.

## Graphical abstract

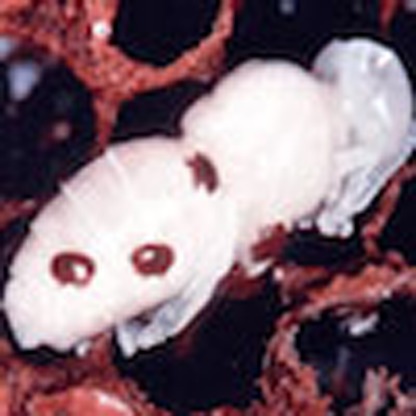

Introductions of novel agents of disease can cause population collapses, creating major conservation problems^1^,^2^,^3^,^4^. In particular, loss of genetic diversity during population bottlenecks can endanger the long-term viability of populations by making them less resilient to future stresses. The dangers posed by introduced infectious diseases are particularly great when they affect agriculturally important species.

The honey bee, *Apis mellifera*, is a prime example of a species that is hugely important to agriculture and has experienced episodes of large-scale mortality caused by new pathogens and parasites. The ectoparasitic mite, *Varroa destructor*, which was introduced from Asia, has killed millions of honey bee colonies in Europe and North America in recent decades^5^. Besides feeding on the haemolymph of adult and developing bees, these mites act as incubators and potent vectors for honey bee viruses, hence, they have fostered the spread of virulent strains of the bees' viruses^6^. Given the importance of intracolonial genetic diversity to disease resistance and other traits of honey bee colonies^7^,^8^, knowing how the massive die-offs caused by *V. destructor* have affected the genetic diversity of honey bees is critical to their conservation and management.

This study investigated the genetic consequences of *V. destructor* on a population of wild honey bee colonies in North America. Prior work has shown that populations of wild colonies in North America experienced crashes coincident with the arrival of *V. destructor*, followed by periods of recovery^9^. Recovery appears to be based partly on immigration of more resistant bees (for example, Africanized honey bees) and partly on selection of relic bees^10^,^11^,^12^.

It is difficult, however, to acquire a full picture of the genetic effects of natural selection on honey bees by studying only present-day samples. Most tools developed thus far for doing so work best at detecting hard selective sweeps^13^,^14^. Moreover, the tools that can detect selection and demographic changes, such as coalescent-based simulation, or approximate Bayesian computation^15^,^16^, are hard to apply to honey bees due to the complexity of their genetics and life history. In particular, honey bees have haplodiploid sex determination and the queens in honey bee colonies mate with numerous drones. Moreover, new colonies are formed by swarming, a process in which the old queen departs with a subset of her workers to establish an additional colony at a new nest site, while one of her daughters becomes the queen and perpetuates the existing colony at the old nest site. Although a colony living in the temperate zone swarms at most a few times a year, it typically produces drones throughout the summer. The many unknown parameters associated with the honey bee's life history make it difficult to accurately generate null expectations of genetic structure.

In principle, the most powerful means of detecting microevolutionary change is to compare allelic frequencies pre and post selection. Museum collections, which provide, a window into the past, offer special opportunities to examine the genetic effects of selection events^17^,^18^. We use voucher specimens collected in 1977 and 2010 from wild honey bee colonies living around Ithaca, NY to take a genome-wide look at the genetic changes that have occurred in honey bees over this 33-year period, which neatly spans the introduction of *V. destructor* to this region of the world. We do so using a novel PCR-free library preparation process developed specifically for this purpose.

We show that while the honey bee population has been hard hit by the introduction of parasitic mites, it most likely did not go extinct. Rather, the bees have rapidly evolved to tolerate the mites' presence, and now exist at the same colony densities today, as they did in the past. This response was most likely polygenic, but possibly involves some of the same pathways previously identified in *Varroa* resistance, namely dopaminergic control of aversive memory. These findings suggest that wild populations of honey bees have an inherent capacity to mount a rapid evolutionary response to novel parasites.

## Results

### Removal of biases

**Figure 1:**
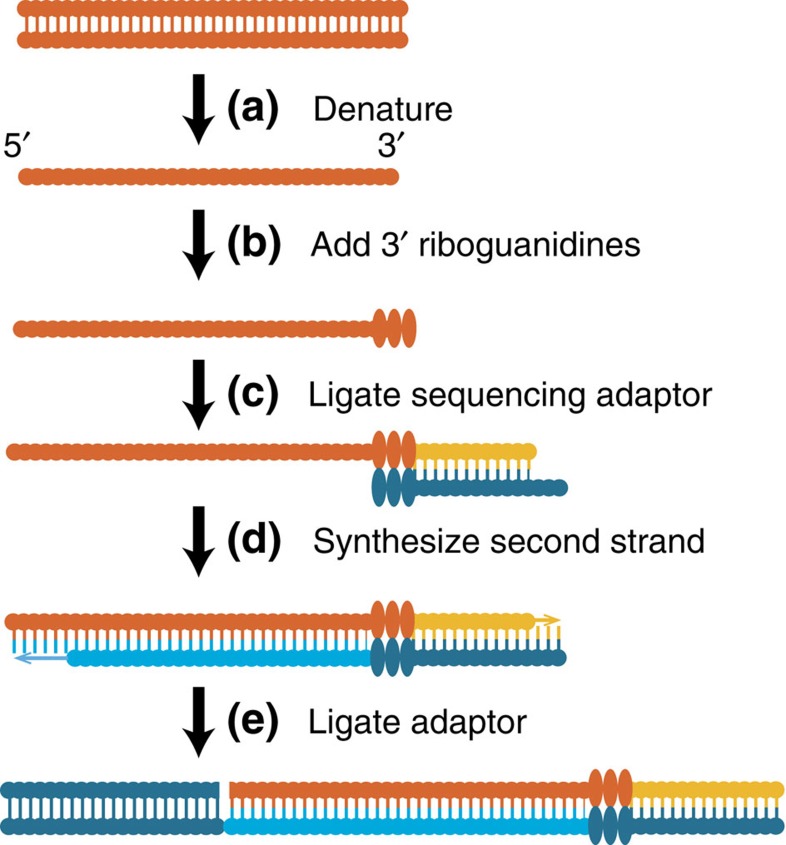
PCR-free library preparation. (**a**) DNA fragments are denatured, and (**b**) a controlled number of riboguanidines are added to the 3′ end^64^.(**c**) This tail was then used to ligate a full-length Illumina sequencing primer, (**d**) which can then be used to synthesize the second strand. (**e**) The other sequencing adaptor can then be ligated to the double-stranded product. This protocol permits direct sequencing of nanogram-scale input templates, without biases and contamination introduced by PCR.

**Figure 2:**
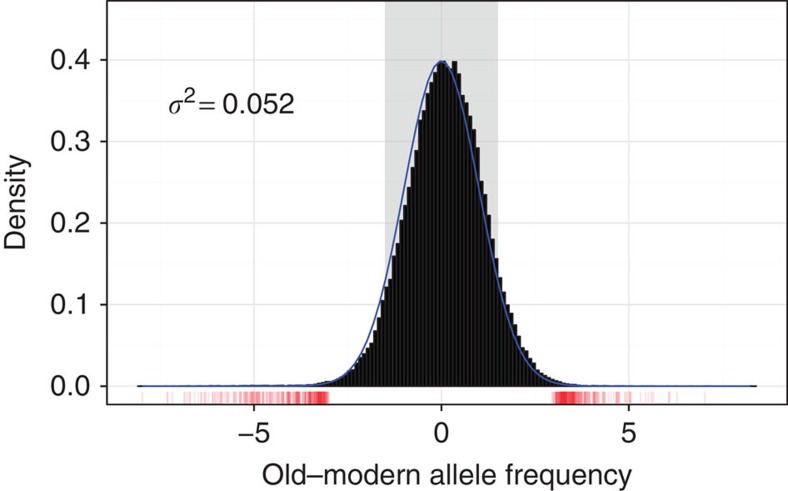
Allelic frequency relationships between old and modern populations for 150,647 SNPs. Allele frequencies were subjected to angular transformation to stabilize variance, following Fisher and Ford^65^. The markers highlighted in red show significant differences between old and modern populations according to both a test based on allele frequencies and a test based on haplotype frequencies. When divided by the s.d., neutral changes in allele frequencies should follow the standard normal distribution (blue line). The 95% confidence interval for possible allele frequency shifts resulting from the joint action of drift and immigration is shown in grey. We used the interquartile range (IQR/1.349) as a robust estimate of the s.d. Allele frequencies should be correlated within a population sampled at various times, scattered around the line *y*=*x* (blue). Points have been rendered transparent to minimize over-plotting. Left over from previous figure version. However, most of the loci are consistent with population genetic expectations for neutrally fluctuating variants.

Museum samples were sequenced using a custom library preparation protocol (Fig. 1). The old and modern specimens differed in degree of DNA fragmentation and postmortem changes in nucleotide composition, which, in turn, created differences in mapping rates and coverage (see Supplementary Table 1 for damage profiles of modern and museum specimens). Short reads from old samples can produce mapping biases, particularly in regions where there are major mismatches to the reference genome sequence, such as in the vicinity of insertion/deletion polymorphisms or multi-nucleotide polymorphisms (MNPs)^19^. It was important, therefore, to conduct stringent filtering, and to verify that the data meet *a priori* expectations of population genetics theory. For instance, the difference between old and modern allele frequencies should be distributed as a random variable with mean zero, and should have a variance related to the number of generations between the two samples, and to the effective population size^20^. The variance-standardized divergences follow a standard normal distribution, suggesting that old and modern specimens give comparable and unbiased estimates of allele frequencies (Fig. 2). Similarly, allele frequencies at neutral loci should be correlated across the two populations, which they are (Supplementary Fig. 1). We conclude that neutral dynamics are sufficiently well captured by our data, allowing us to consider their biological implications. The final SNP density of about one per 1.5 kb prevented us from being able to isolate specific genetic variants that may be direct targets of selection. For historical comparison, the SNP density was higher than other population genomic projects, such as HapMap Phase I^21^, which had a SNP density of about one every 5 kb.

### Estimating the magnitude of immigration and drift

Selection, drift and migration, all cause microevolutionary changes seen as deviations from zero allele frequency differences in Fig. 2. To discuss possible selective effects, we first estimated the magnitude of non-adaptive evolutionary forces. The contribution of immigration and drift to the present-day data can also be evaluated by examining markers absent in the old population, but present in the modern population (12.5% of all markers). Markers absent from the museum population (or present at low frequency) can increase in frequency principally through drift and immigration. As a result, the observed changes in these markers should provide an empirical means for estimating the joint action of these two evolutionary forces over the 33-year period. The change in ‘novel' markers will overestimate the action of drift and migration, since (1) some markers may be absent from museum specimens due to poor read mapping, and (2) some introduced markers may be under positive selection. Both of these factors, and also stochastic sampling variability, will increase the amount of dissimilarity in old and modern allele frequencies, and inflate estimates of changes due to drift and immigration. Despite the conservative estimate of the magnitude of drift and immigration, all alleles showing significant differences between the old and modern populations lie outside the 95% interval where immigration and drift play a significant a role, suggesting instead the action of natural selection (Fig. 2).

### Mitochondrial demographics

**Figure 3:**
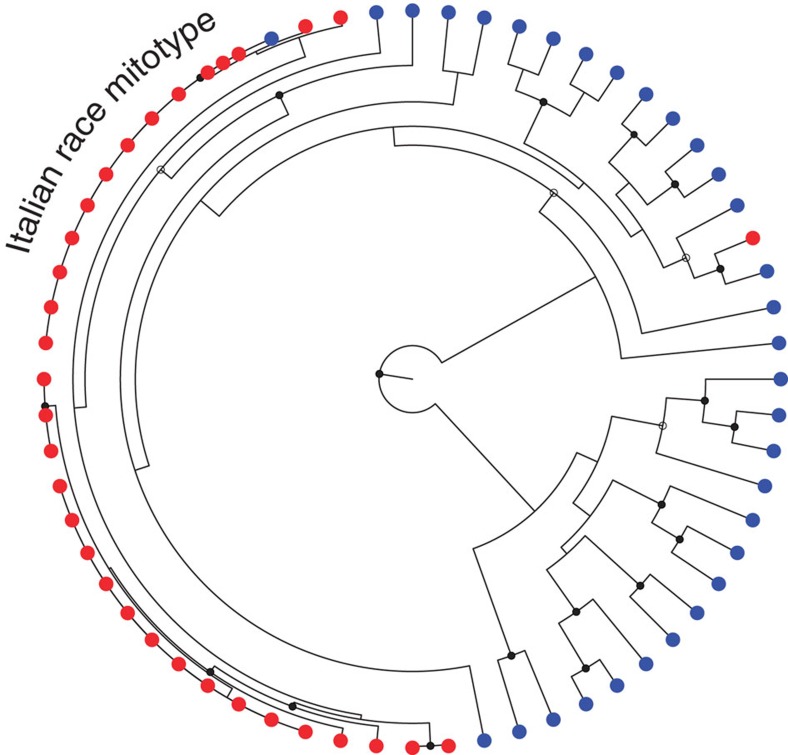
Phylogeny of mitochondrial genomes, and changes in effective population size. The vast majority of mitochondrial genetic diversity in the old population (blue) has been lost in the modern population (red). Coalescent analysis estimated the 95% highest posterior density of the mitochondrial Ne between 2 × 10^5^ and 2 × 10^6^ fifty years ago versus between ∼300 and 1,800 today. These data suggest that the arrival of *V. destructor* was associated with massive colony mortality and intense selection acting on the bees. A major mitochondrial clade appears to have completely disappeared. The most common haplotype present in many of the modern bees and one of the old bees, is identical to the *A. mellifera ligustica* (Italian) mitochondrial haplotype^53^. The modern population appears to have descended from a relatively small number of queens. Nodes with solid circles correspond to 1.0 posterior probability, while those with open circles represents probabilities >0.95.

Demographic history is important for understanding the nature and strength of selection. Unfortunately, no surveys of the wild colonies of honey bees in the study area were conducted in the 1990s, that is, during the arrival of *V. destructor*. To detect a change in population size during that time, we examined changes in the diversity of mitochondrial genomes. Present in many copies, these genomes could be sequenced at extremely high coverage in every sample, producing multi-variant genotypes without missing data, allowing the circumvention of postmortem changes in DNA content through redundant sequence coverage^22^, and the mitochondrial data set was sequenced at extremely high depth (10,481±4,430*x* and 20,422±7,115*x* for old and modern populations, respectively). It consisted of 401 SNPs, and had no missing data. The loss of mitochondrial diversity since 1977 is striking—almost all of the old mitochondrial lineages went extinct, suggesting a massive reduction in effective population size (Fig. 3). Evidently, although the census population size has recovered^23^, most of the surviving bees are descendants of a small number of queens, some of which may have been immigrants that escaped from beekeepers' hives when colonies swarmed.

### Nuclear demographics

**Figure 4:**
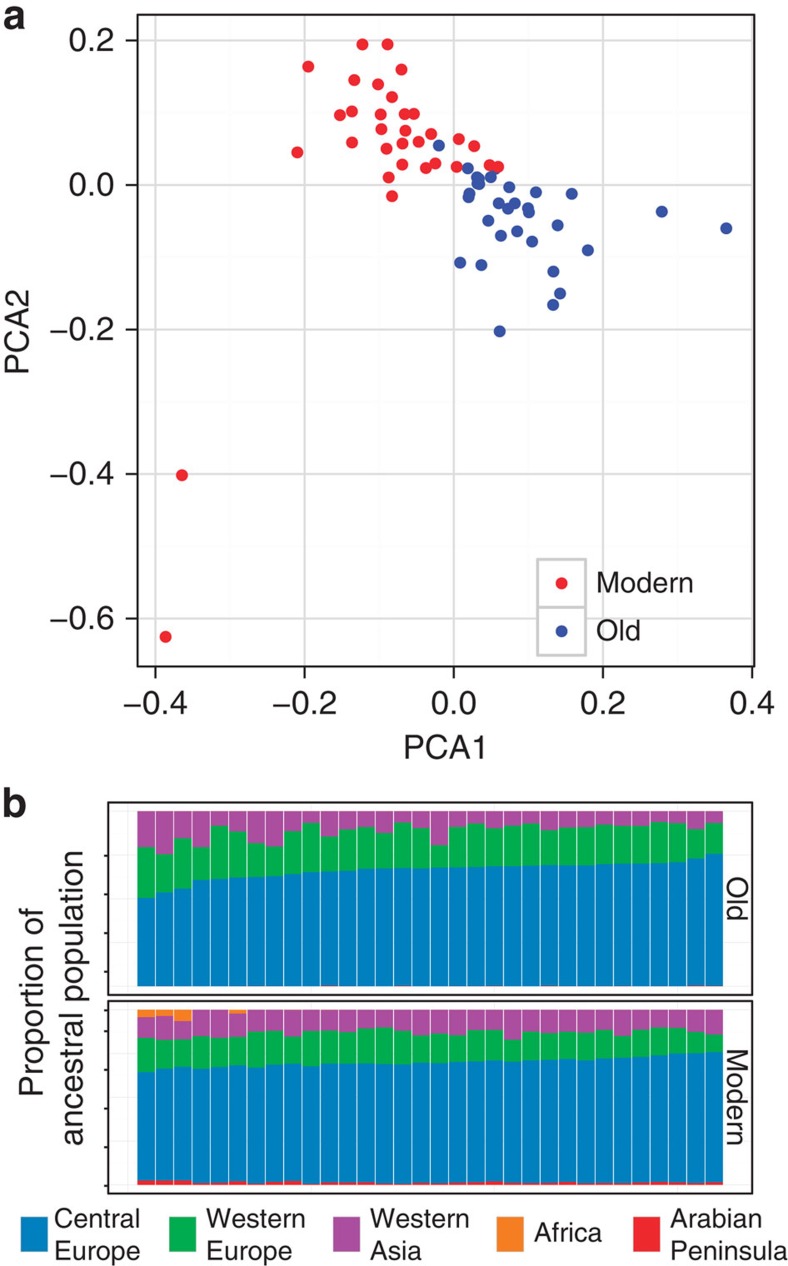
Visualizing nuclear genetic change over time using principal component analysis. (**a**) Principal component analysis and (**b**) changes in ancestral populations. In contrast to mitochondrial data, nuclear data do not show a major reduction in diversity, only a shift in the mean between the two populations. Although the proportions of different ancestral populations have changed, the number of ancestral populations has remained the same. The F_st_ between old and modern populations is 0.022±0.056. This suggests that in spite of colony level mortality caused by *V. destructor*, most of the nuclear genetic diversity has persisted, possibly through production of drones both by strong colonies and by colonies too weak to swarm (produce queens) but still strong enough to produce drones. Admixture analysis suggests that old and modern populations are largely descendants of Central European bees (Carniolan and Italian subspecies popular with beekeepers), though they also have genes from Western Europe and Central Asia. In addition, modern bees have a detectable component of African descent. Interestingly, the modern bees show an increase in genetic ancestry from the Arabian Peninsula, not typically found in domestic stock. These data suggest a mixture of immigration and genetic continuity in the modern population of wild colonies.

Nuclear genomes were sequenced at intermediate coverage for both old and modern populations (8.0±3.5*x* and 16.1±3.5*x*, respectively). After filtering, this data set contained 181,352 high-quality SNP sites (about one per 1.5 kilobases). In contrast to the mitochondrial genome results, no overall loss of nuclear genetic diversity was found (Fig. 4a). Heterozygosities were not different between the two populations (Wilcoxon test *P*=0.070; *n*=168,185 paired sites). Likewise, inbreeding coefficients remained constant (*n*=64, F=0.04±0.03; Welch t_51_=−1.1, *P*=0.25). The lower loss of nuclear, relative to mitochondrial, genetic diversity may result from the combination of extreme polyandry and high outbreeding of honey bees^24^. Taken together, the mitochondrial and nuclear data indicate that the population of wild colonies experienced extremely high mortality, but that this happened without appreciable loss of nuclear genetic diversity.

Biogeographic data from recently published studies^25^,^26^ can be used to examine changes in the composition of ancestral population over time (Fig. 4b; Supplementary Fig. 2). Present-day populations of wild honey bee colonies show a small level of introgression of African genotypes, consistent with the arrival of Africanized bees in the US in the early 1990s. The range of tropically adapted Africanized bees is limited to the southern United States where much of the commercial queen bee production also takes place. The presence of a small number of African genes in New York State most likely results from queen bees being shipped from the southern to the northern states.

### Changes in effective population size

Temporal changes in nuclear allele frequencies can also be used to estimate effective population size (Ne). Variance in differences between allele frequencies in old and modern populations should be equal to the number of generations divided by 2 × Ne^20^. Assuming one or two generations a year, this yields an effective population size between 327 and 653 colonies, respectively. These values may be slight underestimates, as additional variability will be introduced by errors in genotyping^20^, but they are consistent with independent coalescent estimates of Ne for the present-day population from mitochondrial data (Fig. 3). Since Ne is typically about a factor of 10 smaller than the census population size^27^, these results indicate that the population of wild colonies is on the order of several thousand colonies.

### The population of wild honey bees did not go extinct

It is important to consider the possibility that the original population of colonies went extinct following the arrival of *V. destructor* in the study area, and that the current population exists because of immigration of colonies from beekeepers' hives in the surrounding region. A recent microsatellite study of wild and managed honey bee colonies living near Ithaca, NY found strong genetic differences between them, suggesting that the wild population is not merely a sink for escaped domestic colonies^28^. However, there is evidence of gene flow from commercial populations, such as the increase in mitochondrial genotypes commonly used in beekeeping (Fig. 3), and the introgression of African genes (Fig. 4b).

**Figure 5:**
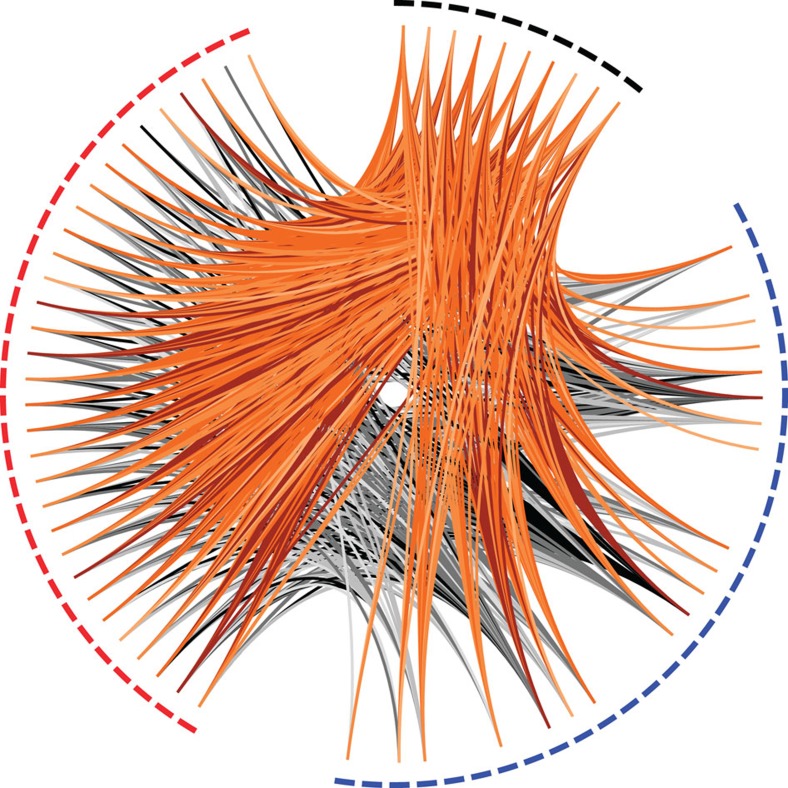
Relatedness of museum and modern specimens collected from wild colonies, relative to a modern specimen collected from domestic colonies. Museum (blue) and modern (red) specimens show asymmetric relatedness relative to an external sample of US domestic bees (black). Museum bees are more closely related to bees from the same populations, than to other US domestic bees (*N*=32, *P*=6.0 × 10^−6^). Similarly, US domestic bees are more closely related to present-day wild bees than they are to museum bees (*N*=10, *P*=2.2 × 10^−3^), suggesting ongoing gene flow into the wild bee population. Links between museum and modern bees are in grey, and links between US domestic bees and Ithaca bees are in orange, with four shades of each colour corresponding to the four quartiles of relatedness greater than zero in the data set.

Two predictions can be made under the scenario that the Ithaca population did not go extinct, but has persisted with some level of immigration. First, because relatedness coefficients decrease exponentially as the degree of relation decreases, museum bees should have more regions identical by descent in common with the present-day Ithaca population than with other US domestic bees with whom they would share more distant ancestry. Second, recent gene flow between modern Ithaca bees and the US domestic bees should create more regions identical by descent between present-day bee populations than between museum bees and US domestic bees. Both these predictions hold true, suggesting that the population has existed continuously (Fig. 5).

### Signatures of selection

The genomic data provide some clues about the mechanisms by which the modern bees tolerate *V. destructor* infestation. Signatures of selection are widespread throughout the genome, with 634 sites showing significant changes in frequency across the two time points (Supplementary Fig. 3). These sites intersected 232 gene models scattered throughout the genome (Supplementary Fig. 3). About half of the enriched gene ontology (GO) terms are related to development, suggesting that resistance may result from changes in the bee's developmental programme (Supplementary Table 2). This seems plausible, given that much of the reproduction by the mites must be completed within the short pupal stage of worker honey bees^29^. Changes in larval movement and moulting patterns might also kill developing mites^30^. Consistent with changes in developmental genes, there were also changes in body size and wing shape.

### Changes in body size and shape

Having found evidence of selection on developmental genes, we predicted that we would find morphological changes over time. Indeed, there has been an overall reduction in body size (head width: *n*=64, t_43.3_=−8.0, *P*=4.0 × 10^−10^; intertegular span: *n*=64, t_62.8_=−8.6, *P*=3.35 × 10^−12^; Supplementary Fig. 5). Likewise, canonical variate analysis of bee-wing landmarks found a significant difference in the mean wing shape between old and modern populations (*n*=64, t_53.4_=11, *P*=2.4 × 10^−15^; Supplementary Fig. 5). These morphological changes are consistent with changes in the underlying developmental programme, though they may also result from stress produced by mite infestation, or other environmental effects. African honey bees, which show resistance to *V. destructor*, are smaller than European honey bees. So the changes in size found here, if adaptive, resulted either from convergent adaptation or perhaps gene flow from honey bees of African descent.

### Comparison with quantitative trait locus studies

Several recent studies have identified quantitative trait loci (QTLs) in bees for a wild population that evolved resistance^31^, or from bees that exhibited behavioural traits that suppress *Varroa*^32^,^33^, making it interesting to examine whether selection acts convergently on resistance mechanisms. It should be noted, that because many genes may be linked to a particular QTL, not just the gene under selection, comparisons have a high potential for false positives, so these results need to be taken with some caution. Nonetheless, there are interesting parallels between previous QTL studies and the evolutionary outcomes of the Ithaca bees. Most strikingly, two of the QTL studies, one focusing on wild honey bees and one focusing on *Varroa*-sensitive hygiene behavior, identified the same dopamine receptor gene (*AmDOP3*), which was also apparently under selection in the Ithaca bees (Supplementary Table 3). This gene has been shown to play a role in aversive memory formation in bees^34^. Two other genes involved in mushroom body development and synapse formation, located within QTL regions on separate chromosomes also show signs of selection in Ithaca (Supplementary Table 3). These genes, and a general enrichment for genes involved in glial cell differentiation (Supplementary Table 2), suggest that behavior plays an important role in dealing with *Varroa* infestation. Overall, the QTL studies support the polygenic nature of adaptation to *Varroa*, as suggested by the wide-ranging genetic changes seen in the Ithaca population of honey bees.

## Discussion

Loss of genetic diversity can render animal and plant populations vulnerable following large-scale collapses caused by new diseases. The genetic changes experienced by honey bees living in the wild outside Ithaca, NY following the introduction of *V. destructor*, which resulted in a severe mitochondrial bottleneck involved little loss of nuclear genetic diversity. This suggests that populations of honey bees living in the wild can survive exposure to novel pathogens. The level of genetic diversity that is present in wild colonies of honey bees may be a good target for managed colonies to assure their future adaptability.

Given the amount of time that passed between the sampling points in this study, genetic changes cannot be ascribed specifically to selection by *Varroa*, since there are likely many selective forces acting in the 33-year period. It seems likely that *Varroa* has caused major genetic changes in the population based on mitochondrial data (Fig. 3), and on comparisons with other populations that were studied immediately after the arrival of mites^11^,^35^. However, some measure of validation comes from finding signs of selection on the *AmDOP3* gene, which appears to be involved in hygienic behavior^32^, in this study, in another *Varroa*-resistant population of bees^31^, and in a QTL study of behavioural resistance to *Varroa*^32^. This gene appears to be a particularly appealing target for marker-based artificial selection aimed at improving bee resistance to mites.

The museum population is more closely related to present-day bees living in the same area compared with other US domestic bees (Supplementary Fig. 4; Fig. 5). However, we cannot strictly rule out the extinction of the Ithaca population followed by re-colonization from more closely related populations including domestic populations as one would have to sample bees from the 1990s when the hypothetical extinction would have taken place. These samples are unlikely to exist. Nonetheless, we think that complete extinction is unlikely for two additional reasons. First, all other wild bee populations that were monitored during the arrival of *Varroa* have gone through major bottlenecks, but none have actually gone extinct^10^,^11^,^35^,^36^. Second, the present-day Ithaca wild bee population maintains mitochondrial genotypes not associated with commercial bee races (Fig. 3), which would be lost in an extinction event. Although these lines of evidence are circumstantial, they all point to continuous persistence of a population in a state of immigration-selection balance.

The findings reported here support the view that responses to sudden changes in selective regimes can be highly polygenic, and can result from changes in allele frequencies of either standing variation or of alleles received through immigration. These signals are not easily detected from present-day data alone. Although studies based on old DNA samples, such as those from museum specimens, are challenging and rarely possible, they provide a valuable look into the past revealing how selection has actually operated in natural populations. Such studies are necessary to develop more sophisticated models of natural selection, ones that consider a wide range of genomic responses together with other features of natural populations, such as demographic change and immigration.

## Methods

### Samples

We used worker honey bees collected from wild colonies living in the forests in Tompkins County near Ithaca, New York, USA (1,233 km^2^). Workers from 32 colonies were collected in 1977 and stored as pinned specimens in the Cornell University Insect Collection^37^. We also used an equal number of workers collected in 2010 from 32 wild colonies living in the same county. Given that drones and queens can fly several kilometres, one can be reasonably sure that the wild bees of Tompkins County form one population^38^. One worker individual per colony was used in the genetic analysis.

### Molecular methods

The overall protocol was similar to that of Tin *et al*.^39^, but with several modifications that allowed the use of a proofreading polymerase and higher yield permitting PCR-free sequencing (Fig. 1). We describe it in detail below, and the most current version of this protocol can be found in our laboratory website (http://ecoevo.unit.oist.jp/site/methods/).

### Genomic DNA extraction

DNA from the museum specimens was extracted as described by Tin *et al*.^39^. The bench top was cleaned with bleach before extraction, then wiped clean with distilled water. Forceps were cleaned with ethanol and flamed before handling specimens. Only guaranteed DNA-free disposable consumables were used and reagents were dedicated to old DNA research only. Consumables and reagents were all used separately from those used in other experiments in the laboratory. Filter tips were used for all experimental procedures. DNA extraction buffer contained 50 g guanidine isothiocyanate, 5.3 ml of 1 M Tris-HCl (pH 7.5), 5.3 ml of 0.2 M EDTA, 10.6 ml of 20% Sarkosyl (IBI Scientific) and 1 ml β-mercaptoethanol, dissolved in 50 ml water (ref. 39). Whole specimens were placed in 2 ml microcentrifuge tubes with 800 μl DNA extraction buffer for an overnight incubation at 55 °C. An equal volume of 99.5% ethanol and 20 μl of silica magnetic beads (Chemicell GmbH) were added to the DNA lysate. Tubes were gently mixed and incubated on a rotary mixer at room temperature for 15 min. All chemicals were purchased from Nacalai Tesque, Inc. unless otherwise stated. Tubes were then placed on a magnetic stand (Invitrogen) for 5 min to separate the supernatant from the magnetic beads. Supernatant that contained proteins and other impurities was discarded. Beads with bound DNA were washed with 200 μl PE buffer (Qiagen) for 10 min at room temperature on a rotary mixer followed by bead separation on a magnetic stand. The washing step was repeated two more times and beads were then air dried for 30–45 min until completely dry. Tubes were then removed from the magnetic stand and DNA was eluted from the beads by resuspending them in 50 μl EB (Qiagen). After 10 min of incubation at 55 °C, DNA was separated from the beads on a magnetic stand and transferred to new microcentrifuge tubes. For modern bees, we extracted genomic DNA using DNeasy blood and tissue kits (Qiagen). The quantity of the DNA was estimated by Quant-iT PicoGreen dsDNA assay kit (Invitrogen). TruSeq low throughput sample preparation kits A and B (Illumina) were used to prepare genomic libraries from modern bees.

### Addition of GTPs to the 3′ termini of genomic DNA

The ribo-tailing reaction requires the presence of an intact 3′-hydroxyl group, which is lost during DNA strand breaks after the formation of abasic sites during DNA degradation^40^. Phosphate groups at the 3′ end of the DNA were removed by treatment with phosphatase. The 13-μl reaction contained 1.3 μl of 10 × buffer 4 (New England Biolabs), 1 μl of 1 U μl^−1^ FastAP thermosensitive alkaline phosphatase (Thermo Scientific) and 200 ng gDNA in EB buffer. The reaction was adjusted with water to reach the reaction volume. The reaction was incubated at 37 °C for 1 h and then heat inactivated at 75 °C for 10 min. We found that there was a significant increase in library yield compared with the same samples without the phosphatase treatment (data not shown).

gDNA was heat denatured into single-stranded DNA at 95 °C for 5 min and then quickly chilled on ice before the ribo-tailing reaction with terminal transferase (TdT; New England Biolabs). The 7-μl TdT reaction consisted of 2.5 μl water, 0.7 μl of 10 × buffer 4, 2 μl of 25 mM cobalt chloride (New England Biolabs), 0.8 μl of 100 mM GTP (Takara), 20 U of TdT and denatured DNA. The 20-μl reaction was incubated at 37 °C for 30 min and then TdT was heat inactivated at 70 °C for 10 min.

### Ligation of Illumina paired-end 2 adaptors

We ligated the following modified Illumina paired-end (PE) 2 adaptor to the riboG-tailed DNA:

- Top strand: 5′-phosphate-AGATCGGAAGAGCGGTTCAGCAGGAATGCddC, ddC=2′,3′-dideoxycytidine-5′-triphosphate
- Bottom strand: 5′-CAAGCAGAAGACGGCATACGAGATCGGTCTCGGCATTCCTGCTGAACCGCTCTTCCGATCT*C*C*CCC, *=phosphorothioate linkage

The 10-μl ligation reaction contained 2.8 μl water, 1 μl of 10 × buffer 4, 3 μl of 10 mM ATP (Sigma-Aldrich), 1.2 μl of 10 μM PE 2 adaptor and 2 μl of 400,000 cohesive end unit per ml T4 DNA ligase (New England Biolabs), which was added to the TdT reaction mixture. The ligation reaction was carried out at room temperature for 90 min, and then heat inactivated at 65 °C for 10 min.

### Second strand DNA synthesis with a proofreading polymerase

The 10-μl reaction consisted of 0.68 μl water, 8 μl of 5 × HF buffer (Thermo Scientific), 0.32 μl of 25 mM dNTP (Promega) and 1 μl of 2 U μl^−1^ phusion high-fidelity DNA polymerase (Thermo Scientific), which was added to the ligation reaction. The reaction was incubated at 72 °C for 45 min (optional stop point: you may freeze the samples at −20 °C at this point and continue the procedures the next day). Dephosphorylation was performed to remove the phosphate groups of excess PE 2 adaptors in the reaction by the addition of 20 U calf-intestinal alkaline phosphatase (Invitrogen). The reaction was carried out at 37 °C for 1 h. 20 U was exactly what we used, 20 U of calf-intestinal alkaline phosphatase were added to the reaction and incubated at 50 °C for 1 h to maximize the phosphatase activity at the 5′ recessed end of the adaptors. The reactions were then purified with MinElute reaction cleanup kit (Qiagen) and eluted in 10 μl EB buffer.

### Ligation of barcoded Illumina PE 1 adaptor to the double-stranded DNA

The library was constructed by ligation of double-stranded DNA fragments with barcoded Illumina PE 1 adaptors:

- Top strand: 5′-CGACGCTCTTCCGATCTxxxxxxddC
- Bottom strand: 5′-phosphate-GxxxxxxAGATCGGAAGAGCGTCGTGTAGGGAAAGAGTGTAGATCTCGGTGGTCGCCGTATCATT, xxxxxx=barcode

The 11-μl reaction consisted of 5 μl of 2 × Quick ligation buffer (New England Biolabs), 0.5 μl of 50 μM adaptor, 1 μl of 2,000,000 cohesive end units per ml T4 DNA ligase (New England Biolabs) and 4.5 μl of purified DNA. Non-specific ligation was greatly reduced with the use of 3′-ddC adaptor. The ligation reaction was carried out at 16 °C overnight.

Ligation products were purified by solid-phase reversible immobilization after (Tin *et al*.^39^) using Dynabeads MyOne carboxylic acid (Invitrogen). Dynabeads MyOne carboxylic acid was washed twice in EB buffer and then resuspended in the same volume of EB buffer. Polyethylene glycol (PEG) 6000 was dissolved in autoclaved Milli-Q water to a 40% w/v solution and then filter sterilized through a 0.22-μm filter into a sterile container (Corning). The 40% PEG was diluted to 19% with water, with a final concentration of 0.9 M NaCl and 10 mM Tris-HCl, pH 6. Ligation reactions were adjusted to 50 μl final volume with water before purification. Approximately, 100 μl of 19% PEG/NaCl/Tris and 10 μl of prepared Dynabeads were added to each ligation reaction and resuspended. The mixture was incubated at room temperature for 5 min. Tubes were then placed on a magnetic stand for 5 min. Supernatant was discarded and beads were washed twice with 70% ethanol (with 10 mM Tris, pH 6 in final concentration), and dried for 5–10 min at room temperature. The beads turn from dark brown to light brown when they are dry. Overdry will cause DNA to be difficult to elute from the beads and will cause loss of yield. Tubes were then taken off the magnetic stand and bound DNA was eluted from the beads by resuspending them in 10 μl EB buffer. After 5 min of incubation at room temperature, beads were separated on the magnetic stand and the eluent containing the library was saved. The concentration of the libraries was measured with quantitative PCR (Kapa Biosystems). Equimolar of libraries were pooled with different barcodes (5′-GAGGAT-3′, 5′-GTCCAA-3′, 5′-AGATT-3′, 5′-ATCAC-3′, 5′-TCAT-3′ and 5′-CGT-3′). The pooled library size distribution was checked using the Bioanalyzer High Sensitivity DNA kit (Agilent Technologies) before sequencing.

### Library sequencing

Genomic libraries from old honey bee were sequenced using 58 and 110 bp single-end reads (one and three flow cells, respectively). Libraries made from modern bees were sequenced in 2 × 100 bp mode on a single flow cell in an Illumina Hiseq 2000. DNA sequencing was performed at core facility of the Okinawa Institute of Science Technology.

### Bioinformatic analysis

The entire bioinformatic pipeline starting from genotype calling and intermediate analysis files can be found on DataDryad (http://dx.doi.org/10.5061/dryad.vn607). We briefly summarize the pipeline below. We used the Amel_4.5 genome assembly and the OGSv3.2 gene set for the analysis^41^. GO terms for the gene set were inferred using BLAST2GO^42^ based on blastx results against The National Center for Biotechnology Information (NCBI's) nr database (e-value cutoff 10^−3^). Raw reads from modern data were mapped using bowtie2 in end-to-end mode^43^ (default parameters). Short reads were sorted by barcode and adaptor trimmed using a custom script, and were mapped using Stampy (v 1.0.23)^19^ (—sensitive). Initial variant discovery was performed using FreeBayes^44^ (default parameters, but —theta 0.002, to account for higher variability in bees, see *call.sh*), and estimation of allele frequencies was carried out using ANGSD^45^ (see the file *angsd.sh* code repository for details). Inbreeding coefficients were estimated as in Vieira *et al*.^46^, and were then used as priors for Fst estimation^47^ (see *angsd_inbreeding.sh* and *angsd_ngsFst.sh* in the code repository). The file *pca_all.sh* contains details of the principal component analysis^47^. Analysis of ancestral population composition was performed using NGSAdmix (v. 32)^48^ using default settings. NGSAdmix assigns individuals probabilistically to one of *K* hypothetical populations, using the same model as STRUCTURE^49^, but accounting for genotype uncertainty (see *ngsAdmix.sh*). In this analysis, we used our own data and previously published data sets^25^,^26^. We ran this analysis for *K*=5, which was a level of granularity sufficient to capture major patterns in worldwide honey bee population structure^26^. For all analyses, except those of mitochondrial population genetics and relatedness estimation, we performed tests without calling individual genotypes, a strategy that alleviates biases due to coverage differences between populations for allele frequency estimation and measurements of population differentiation^47^,^50^. These methods employ probabilistic frameworks accounting for variability in coverage, as well as considering mapping and sequencing errors to compute overall ‘genotype likelihoods', which are used in higher-order computations.

Relatedness between individuals from museum, modern and a present-day US domestic population, was inferred using method of Yang *et al*.^51^ implemented in VCFTools^52^. We used relatedness link degrees (the number of individuals sharing positive relatedness with a focal individual) as a measure of relatedness between populations. The statistical significance of the link degrees was tested using paired *t*-tests. Because there were only 10 samples from the present-day US domestic population available, versus 32 in the modern bee sample, the link degrees to these to populations from the museum specimens would be unequal. Therefore, when testing the statistical significance of the relatedness of museum samples to these two populations, we randomly chose 10 modern Ithaca bee samples for the comparison.

### Data filtering

The data set was stringently filtered at the site level to minimize biases. Because each read has less information, and possible mutations due to degradation, short single-end reads from museum specimens map less effectively than long paired-end reads from modern material, particularly in areas of genomic variability. As longer genetic variants are more difficult to map, we focused our analysis on SNPs to minimize mapping biases. We also removed any SNP within 25 bases of another variant from the analysis, as changes at one of these sites had the potential to affect mapping rate at the other. We also removed any SNPs lying within repetitive regions. We also applied filters removing sites with quality scores <60, >30% overall missing data. For most analyses, except those estimating strength of migration (see section ‘Estimating strength of genetic drift and migration' below), we also removed sites with <10% minor allele frequency. These filters resulted in an increased correlation between allele frequencies in old and modern data. The caption to Supplementary Fig. 1 provides a detailed analysis of the effect of filtering on the extent of bias, and a discussion of possible cytosine deamination artefacts.

### Coalescent analysis of mitochondrial DNA

Reads were mapped to the mitochondrial reference^53^. In addition to FreeBayes, we used GATK's UnifiedGenotyper to call SNPs and indels^54^. The two data sets were filtered to remove low quality sites (<20), indels, sites with missing data and intersected using BedTools^55^ to create a reference SNP panel. Resulting variant calls were converted to DNA sequence and used for coalescent analysis using BEAST^56^, after partitions and corresponding nucleotide substitution models were determined by PartitionFinder^57^. We ran the analysis for 10^7^ generations with convergence and adequate estimated sample size (>100 for important parameters) using Tracer (v 1.6)^56^. This BEAST analysis was repeated three separate times to confirm that the solution was stable. The XML file with the run parameters is included in the DataDryad repository.

### Body size measurement

Worker body size was assessed by measuring head width and intertegular span, both reliable methods for determining overall body size^58^. Head width was defined as the greatest diameter, and was measured crosswise through the middle of the eyes in front view. Intertegular span is the distance between the tegulae of the thorax. Specimen images were taken by stereo microscope Leica M205C at × 20 and × 16 for head and thorax measurement. Measurements were performed using ImageJ version 1.47.

### Analysis of wing shape

Both forewings of each bee were dissected and mounted between microscope glass slides. Subsequently, they were photographed using a Nikon SMZ1500 at × 10 magnification. Coordinates of 19 landmarks at junctions of wing veins were digitized using a pen and tablet system (Wacom Bamboo). Raw coordinates were subjected to Procrustes superimposition. Both data digitization and Procrustes analysis were conducted by using the CLICS software package^59^. principal components analysis (PCA) analysis based of the population means' covariance matrix was performed in the Morpho package^60^ in R^61^.

### Detecting allele frequency changes

To identify alleles that differ significantly between the old and modern population, we used a maximum likelihood estimator that does not infer genotypes for any one individual^50^. This same framework also allows likelihood ratio testing that accounts for uncertainty in the observed genotypes for association mapping. Both calculations were performed using ANGSD (0.533)^45^. Likelihood ratio testing results were converted to *P* values, assuming a *χ*^2^ distribution with one degree of freedom. *P* values were adjusted for multiple comparisons using Benjamini and Hochberg^62^ correction for multiple comparisons, with the family-wise error rate set at 5%.

### Estimating strength of genetic drift and migration

We focused on alleles that were apparently absent from the old population, many of which were present at low frequencies and were filtered from the main analysis. These alleles were probably introduced into the Ithaca population by migration between 1977 and 2010, and have increased in frequency either by selection or in most cases by drift. We then estimated the 95% confidence interval for the frequency change in alleles absent from the old populations using non-parametric bootstrap. This is a conservative estimate, since some alleles were absent from the old population may be in the regions where degraded DNA did not map well; including such regions would inflate the apparent effect of drift.

### GO term enrichment

We used the GOstats package to conduct hypergeometric tests of GO term enrichment^63^. Genes in SNP-rich regions and longer genes were more likely to have significant SNPs detected. Therefore, we computed a null distribution of GO terms by permuting the positions of the selected SNPs and conducting a separate hypergeometric test for all of them. Only GO terms that were present in the original data set and in ≤5% of the simulated data sets were retained.

## Additional information

**Accession codes**: DNA sequencing data generated in this study have been deposited in GenBank/EMBL/DDBJ as BioProject PRJDB3198.

**How to cite this article:** Mikheyev, A. S. *et al*. Museum samples reveal rapid evolution by wild honey bees exposed to a novel parasite. *Nat. Commun.* 6:7991 doi: 10.1038/ncomms8991 (2015).

## Supplementary Material

Supplementary Information Supplementary Figures 1-5, Supplementary Tables 1-3 and Supplementary References
