# Supplementary material for: Museum samples reveal rapid evolution by wild honey bees exposed to a novel parasite
Source: Nat Commun. 2015 Aug 6;6:7991. doi: 10.1038/ncomms8991 (PMC4918369; doi:10.1038/ncomms8991)
Supplement: Supplementary Information — Supplementary Figures 1-5, Supplementary Tables 1-3 and Supplementary References [file ncomms8991-s1.pdf]

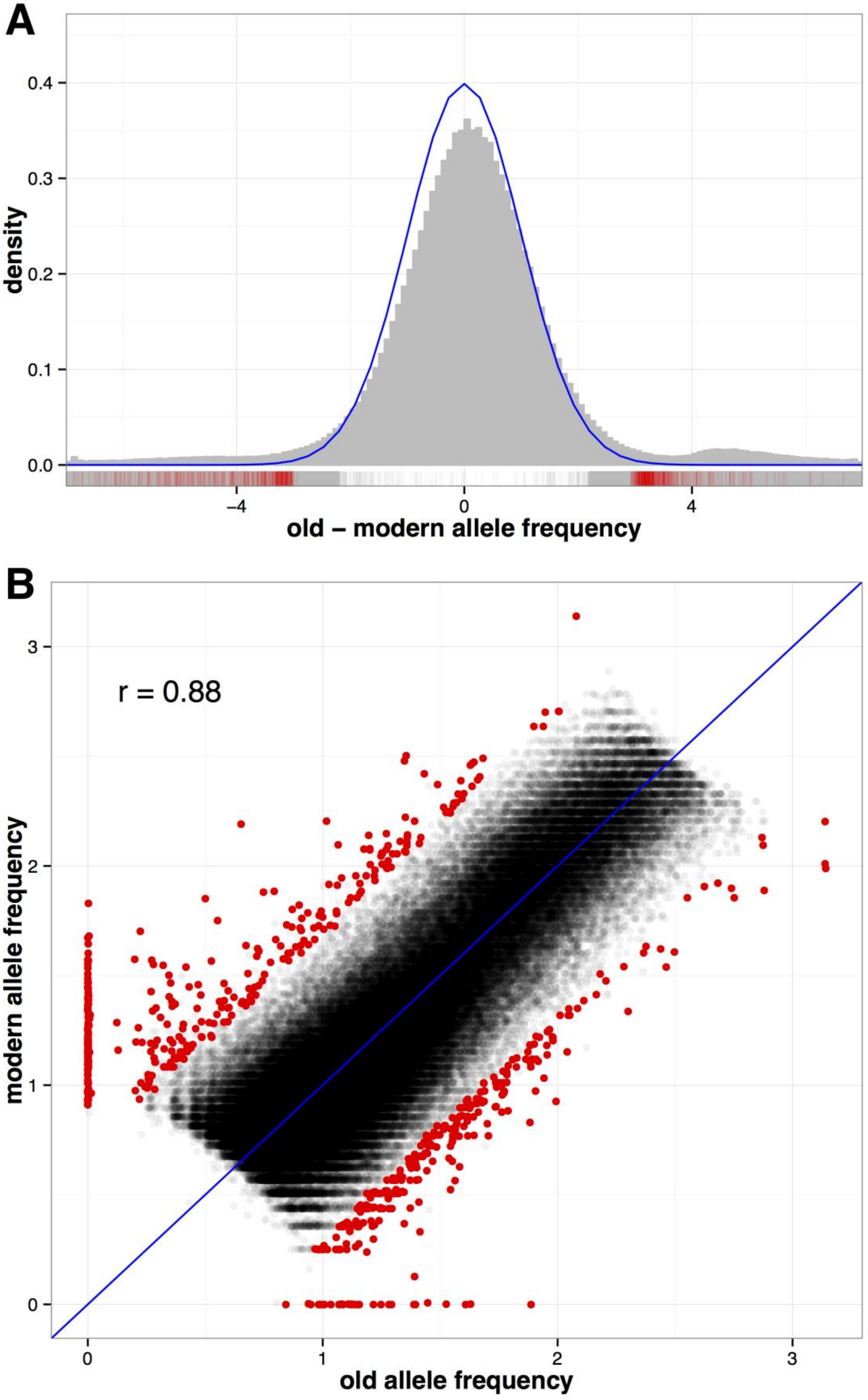
A
0.4
0.3
0.2
0.1
0.0
density
−4
0
4
old – modern allele frequency
B
r = 0.88
3
2
1
0
modern allele frequency
0
1
2
3
old allele frequency

Supplementary Fig. 1. Extensive quality control is necessary prior to interpretation of population genomic data comparing modern and museum specimens. (A) With only technical quality control filtering, the distribution of differences between old and modern samples strongly deviates from the standard normal distribution (blue line), with heavy tails corresponding to extreme differences, which all appear significant under likelihood ratio tests (grey lines in the rug plot below the main plot). Although it is tempting to ascribe them to biological factors, they largely disappear after additional quality filtering (Figure 2). The following quality filters applied in the top panel: minimum site quality score 60, maximum two alleles, 30% maximum missing data per site, no indels, 10% minimum minor allele frequency (vcftools --minQ 60 --max-alleles 2 --max-missing 0.7 --remove-indels --maf 0.1). (B) After additional filtering to account for potential mapping biases the distribution is much more close to normal (also see Figure 2). A blue line shows the null expectation y=x, and red points indicate SNPs that show significant differences in the two populations, and correspond to red lines in the rug plot in the top graph. Alleles along the y-axis, which correspond to alleles missing from the old population, provide evidence of immigration. However, most of the loci are consistent with population genetic expectations for neutrally fluctuating variants. Old and modern allele frequencies show a high level of correlation, compared to unfiltered data (r = 0.69), suggesting that these additional filters improve data quality. Allele frequencies were subjected to angular transformation, as in Figure 2.

We can more specifically allele frequencies of at sites we expect to be most affected by postmortem damage, such as cytosine deamination, which causes C -> T mutations, are biased in the two populations. There were no differences in allele frequencies at C/T SNP sites in the old and modern populations, suggesting that they are not (one-sample t-test t=0.14, d.f. = 77455, p = 0.89, mean = $5.6*10^{-5}$). There were also no false positive SNP sites, *i.e.,* sites that were fixed for a cytosine in a modern population, but polymorphic for cytosines and thymines in the museum populations.

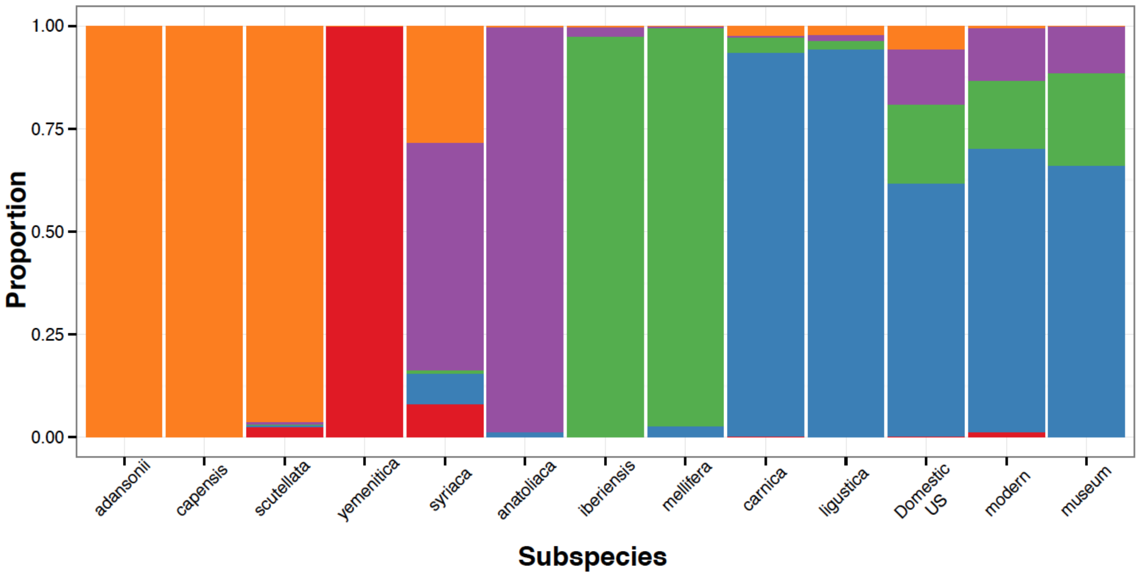

Supplementary Fig. 2. Genetic structure of worldwide bee populations. Each subspecies or population can be a member of up to five ancestral populations[1,2]. Domestic bee populations in the US, have a significantly larger African contribution than their wild counterparts. Interestingly, the amount of Arabian genetic ancestry, as in the yemenitica subspecies, which is virtually entirely absent in managed bee stock, has also slightly increased post-varroa.

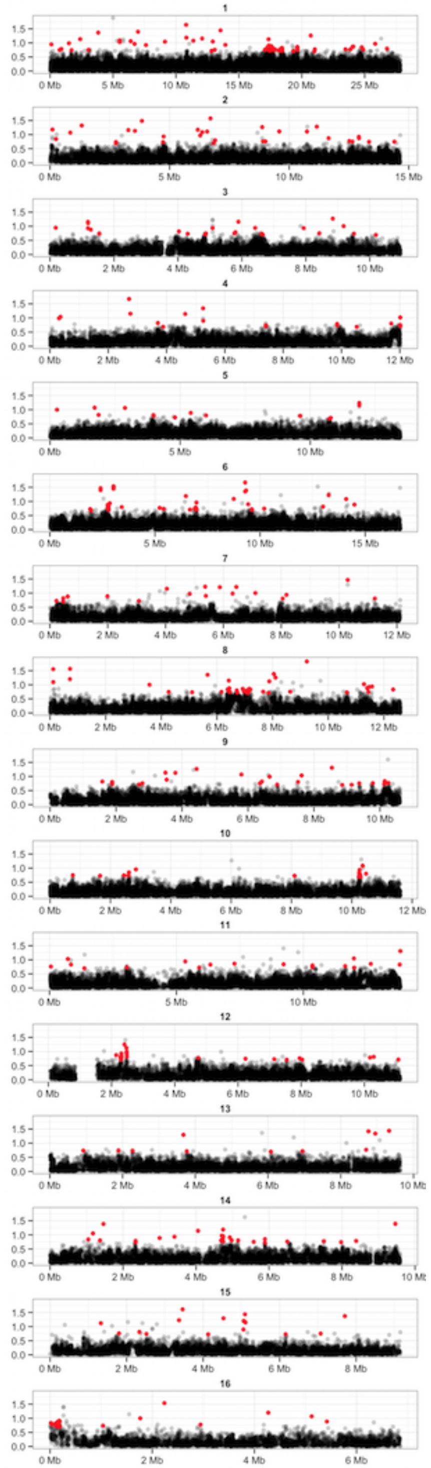

Supplementary Fig. 3. Sites under selection are widely distributed throughout the genome. Most site that differed significantly in frequency between old and modern populations is surrounded by SNPs that were not significant.

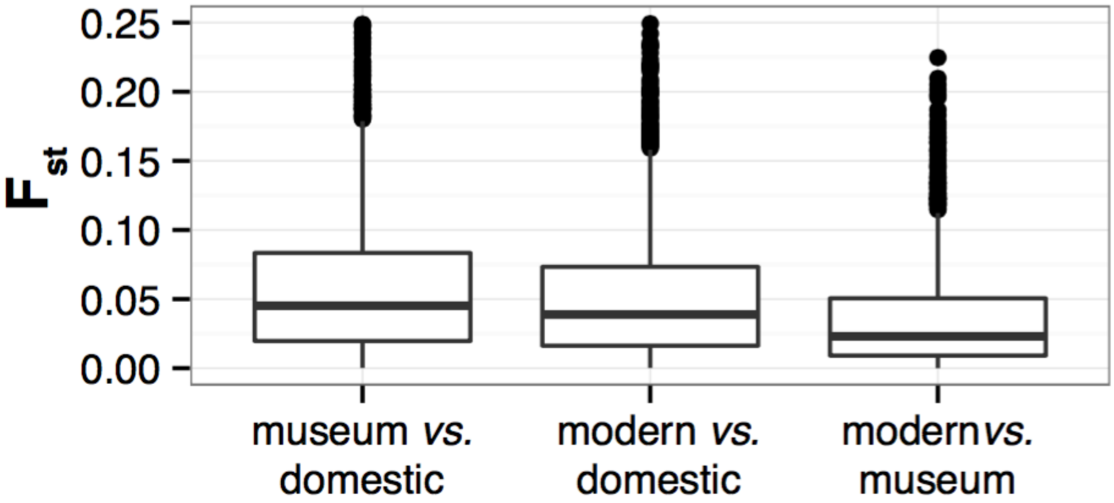

Supplementary Fig. 4. Genome-wide F_{st} differences show that modern and museum bees are more closely related to each other than either is to other domestic bees. The plot shows F_{st} values > 0, and does not show outliers above F_{st} 0.25 for legibility. All differences are statistically significantly significant (N = 95,099 sites, Krukal-Wallis < 0.001). These results complement the analysis summarized in Figure 5, both suggesting that there was genetic continuity between modern and museum populations.

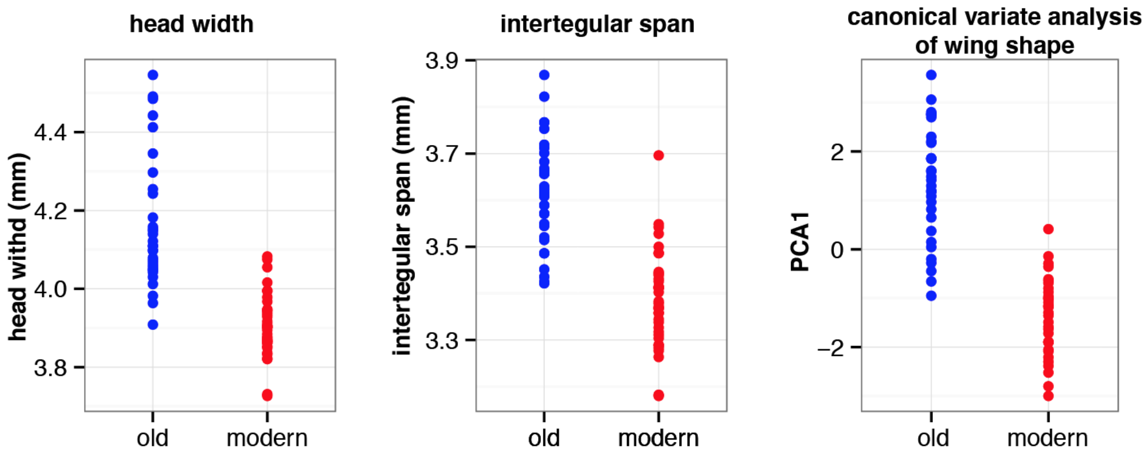

Supplementary Fig. 5. Morphometric analysis of old and modern populations. The two populations were significantly different in two body size measures (head width and intertegular span). They also differed in overall wing shape, as measured by 19 wing landmarks[4].

Supplementary Table 1. Sequencing depth and data content of museum and modern samples. All modern samples were sequenced in paired end mode, while old samples were sequenced in single end mode.

sample	pop.	reads	mapping	total bases	coverage	accession
**HB01**	modern	45,863,698	92%	4,266,915,165	18.62	DRX028452
**HB02**	modern	48,173,262	94%	4,539,885,424	19.82	DRX028453
**HB03**	modern	46,167,248	93%	4,328,421,555	18.89	DRX028454
**HB05**	modern	40,196,854	92%	3,743,183,528	16.34	DRX028455
**HB06**	modern	68,706,632	94%	6,502,346,778	28.38	DRX028456
**HB07**	modern	49,757,004	94%	4,722,202,721	20.61	DRX028457
**HB08**	modern	31,661,544	93%	2,978,087,543	13	DRX028458
**HB09**	modern	29,053,926	93%	2,715,614,555	11.85	DRX028459
**HB10**	modern	37,940,650	92%	3,500,829,058	15.28	DRX028460
**HB11**	modern	27,657,214	94%	2,607,066,245	11.38	DRX028461
**HB12**	modern	38,077,920	93%	3,569,974,918	15.58	DRX028462
**HB13**	modern	31,338,982	93%	2,924,376,455	12.76	DRX028463
**HB14**	modern	37,557,060	91%	3,444,784,469	15.04	DRX028464
**HB15**	modern	33,879,484	94%	3,199,535,365	13.97	DRX028465
**HB16**	modern	44,324,050	94%	4,205,141,602	18.35	DRX028466
**HB17**	modern	33,483,408	92%	3,093,584,277	13.5	DRX028467
**HB18**	modern	37,851,690	92%	3,516,033,248	15.35	DRX028468
**HB19**	modern	44,734,688	92%	4,131,013,722	18.03	DRX028469
**HB20**	modern	36,745,526	90%	3,320,312,629	14.49	DRX028470
**HB23**	modern	46,106,772	73%	3,394,185,882	14.81	DRX028471
**HB25**	modern	38,910,218	93%	3,662,254,117	15.99	DRX028472
**HB26**	modern	36,583,138	93%	3,440,653,170	15.02	DRX028473
**HB27**	modern	51,257,616	94%	4,839,236,055	21.12	DRX028474
**HB28**	modern	43,592,298	92%	4,033,914,572	17.61	DRX028475
**HB29**	modern	43,124,590	93%	4,024,555,688	17.57	DRX028476

**HB30**	modern	32,103,394	91%	2,944,162,039	12.85	DRX028477
**HB31**	modern	32,897,002	92%	3,043,939,795	13.29	DRX028478
**HB32**	modern	44,922,232	93%	4,186,580,705	18.27	DRX028479
**HB33**	modern	36,039,152	92%	3,336,733,345	14.56	DRX028480
**HB34**	modern	38,594,938	94%	3,656,652,708	15.96	DRX028481
**HB35**	modern	27,347,424	93%	2,558,597,560	11.17	DRX028482
**HB36**	modern	38,814,880	93%	3,639,275,450	15.88	DRX028483
**Box_10a**	old	56,716,652	46%	1,438,933,464	6.28	DRX028523
**Box_11a**	old	53,770,010	73%	2,232,717,618	9.75	DRX028524
**Box_13b**	old	45,583,306	34%	856,748,576	3.74	DRX028525
**Box_14b**	old	58,123,290	79%	2,443,680,173	10.67	DRX028526
**Box_15b**	old	45,452,579	63%	1,355,037,226	5.91	DRX028527
**Box_16a**	old	66,570,832	33%	1,362,204,905	5.95	DRX028528
**Box_17b**	old	48,883,105	83%	2,042,252,628	8.91	DRX028529
**Box_18a**	old	47,831,199	93%	2,416,503,968	10.55	DRX028530
**Box_1a**	old	48,936,905	78%	2,022,508,487	8.83	DRX028522
**Box_3b**	old	43,257,076	75%	1,536,273,154	6.71	DRX028531
**Box_4b**	old	69,669,529	18%	708,511,168	3.09	DRX028532
**Box_5a**	old	65,341,301	28%	1,105,460,881	4.83	DRX028533
**Box_6b**	old	34,707,658	40%	899,880,319	3.93	DRX028534
**Box_7b**	old	54,597,692	71%	2,093,673,822	9.14	DRX028535
**Box_8a**	old	49,498,166	82%	2,087,940,454	9.11	DRX028536
**Box_9a**	old	48,624,590	84%	2,239,642,462	9.78	DRX028537
**Tree_10a**	old	64,417,102	92%	3,244,878,999	14.16	DRX028539
**Tree_11a**	old	66,131,942	88%	3,395,776,078	14.82	DRX028540
**Tree_12a**	old	22,438,591	38%	424,007,448	1.85	DRX028541
**Tree_12b**	old	35,675,331	79%	1,418,418,364	6.19	DRX028542
**Tree_13b**	old	48,727,107	93%	2,328,880,337	10.17	DRX028543
**Tree_14b**	old	45,169,343	91%	2,053,770,963	8.96	DRX028544

**Tree_1b**	old	50,504,667	73%	1,895,294,695	8.27	DRX028538
**Tree_2b**	old	66,513,206	91%	3,203,882,913	13.98	DRX028545
**Tree_3a**	old	73,722,652	29%	1,317,844,140	5.75	DRX028546
**Tree_4a**	old	63,393,204	51%	1,911,439,428	8.34	DRX028547
**Tree_5b**	old	37,911,859	36%	657,175,716	2.87	DRX028548
**Tree_6a**	old	35,079,555	83%	1,438,994,485	6.28	DRX028549
**Tree_6b**	old	75,523,483	86%	3,774,732,074	16.48	DRX028550
**Tree_7b**	old	52,994,680	69%	1,949,276,934	8.51	DRX028551
**Tree_8a**	old	41,610,297	54%	1,307,696,193	5.71	DRX028552
**Tree_9a**	old	54,977,892	56%	1,749,804,494	7.64	DRX028553

Supplementary Table 2. Biological process GO terms enriched among genes that significantly changed in frequency. Because longer and more SNP-rich gene models have a higher chance of showing signs of selection, a null model was computed by permuting detected SNPs 1000 times. A separate hypergeometric GO term enrichment analysis was carried out for each permutation and the original data. GO terms enriched in the original data, but not in the permuted samples are presented below, with p-values corresponding to their frequency in the permuted data. Four of the eight enriched terms (GO:0035321, GO:0042249, GO:0060297,GO:0010001) are involved in development, suggesting that resistance to mites may result from changes to larval growth morphology, tempo, or some other ontogenetic processes that reduce the mites' growth rates. Changes in body size and shape are consistent with these genetic changes (Figure S3). One GO term is associated with neural function, which parallels the genes associated with neurogenesis and behavior identified by QTL studies (Supplementary Table 3).

**ID**	**Description**	**p-value**
GO:0007043	cell-cell junction assembly	0.011
GO:0043297	apical junction assembly	0.011
GO:0060297	regulation of sarcomere organization	0.02
GO:0035321	maintenance of imaginal disc-derived wing hair orientation	0.027
GO:0010800	positive regulation of peptidyl-threonine phosphorylation	0.032
GO:0042249	establishment of planar polarity of embryonic epithelium	0.032
GO:0010001	glial cell differentiation	0.04

Supplementary Table 3. Overlap between genes showing significant allele frequency changes in the Ithaca population that were also in regions with QTL markers linked to Varroa resistance in other studies. Because QTL regions include loci under selection, as well as genes immediately linked to them, intersecting gene lists is imperfect and will generate many false positives. However, GB14561 was found to play a role in two previous QTL studies and is under selection in the Ithaca population, suggesting it plays a general role[5,6]. Other genes, such as GB11239 and GB19232 are also involved in neurogenesis and behavior

**honey bee gene id**	***Drosophila* homolog id**	**prediction**	**putative function**
GB15278[5]	CG42402	hypothetical protein LOC724835	
GB14379[5]	CG15020	hypothetical protein LOC725078	
GB14561[5,6]	CG33517	Dop3 D2-like dopamine receptor	aversive olfactory learning
GB13565[5]		inositol hexakisphosphate kinase 2-like DUF2475 superfamily	protein phosphorylation, phosphatidylinositol metabolic processing
GB19232[5]	CG17221	reticulon-4-interacting protein, mitochondrial-like; MDR superfamily; AdoMet_MTases superfamily	mushroom body development
GB11239[7]		Wnt-7b-like	Wnt signalling pathway
GB18754[7]	CG7050	Neurexin 1 EGF_CA and LNS superfamily domains	Synapse initiation, maintenance and function of synapses

Supplementary References

1. Wallberg, A. *et al.* A worldwide survey of genome sequence variation provides insight into the evolutionary history of the honeybee *Apis mellifera*. *Nat Genet* **46,** 1081–1088 (2014).
2. Harpur, B. A. *et al.* Population genomics of the honey bee reveals strong signatures of positive selection on worker traits. *Proc. Natl. Acad. Sci. U.S.A.* **111,** 2614–2619 (2014).
3. Manichaikul, A. *et al.* Robust relationship inference in genome-wide association studies. *Bioinformatics* **26,** 2867–2873 (2010).
4. Francoy, T. M. *et al.* Identification of Africanized honey bees through wing morphometrics: two fast and efficient procedures. *Apidologie* **39,** 488–494 (2008).
5. Tsuruda, J. M., Harris, J. W., Bourgeois, L., Danka, R. G. & Hunt, G. J. High-resolution linkage analyses to identify genes that influence Varroa sensitive hygiene behavior in honey bees. *PLoS ONE* **7,** e48276 (2012).
6. Behrens, D. *et al.* Three QTL in the honey bee Apis mellifera L. suppress reproduction of the parasitic mite *Varroa destructor*. *Ecol Evol* **1,** 451–458 (2011).
7. Arechavaleta-Velasco, M. E., Alcala-Escamilla, K., Robles-Rios, C., Tsuruda, J. M. & Hunt, G. J. Fine-scale linkage mapping reveals a small set of candidate genes influencing honey bee grooming behavior in response to Varroa mites. *PLoS ONE* **7,** e47269 (2012).
